# Optimizing the Compressive Properties of Porous Aluminum Composites by Varying Diamond Content, Space Holder Size and Content

**DOI:** 10.3390/ma16030921

**Published:** 2023-01-18

**Authors:** Bisma Parveez, Nur Ayuni Jamal, Md Abdul Maleque, Alya Naili Rozhan, Abdul Aabid, Muneer Baig

**Affiliations:** 1 Department of Manufacturing and Materials Engineering, Kulliyyah of Engineering, International Islamic University Malaysia, Kuala Lumpur 53100, Malaysia; 2 Department of Engineering Management, College of Engineering, Prince Sultan University, Riyadh 11586, Saudi Arabia

**Keywords:** porous aluminum composite, relative density, porosity, Taguchi L9 orthogonal array plateau stress, energy absorption capacity

## Abstract

The compressive properties of powder metallurgy (PM)-based porous aluminum (Al) composites were optimized at three levels based on the following parameters: titanium (Ti)-coated diamond content, polymethylmethacrylate (PMMA) particle content, and PMMA particle size. A 3 × 3 matrix was used in the experimental design of an L9 orthogonal array to get nine sets of combinations. These nine compositions were then tested and analyzed for density, porosity, plateau stress, and energy absorption capacity. The effect of individual input parameters was assessed using the Taguchi-based means ratio and analysis of variance (ANOVA). The main effect plots articulated the optimal parameter levels for achieving maximum compressive property values (plateau stress and energy absorption capacity). The findings show that diamond content and PMMA particle size have a major impact on compressive properties. The ANOVA analysis yielded similar results, with diamond content accounting for the greatest value. Further, the response optimization of compressive properties revealed that maximum values could be obtained at optimum parameters: diamond content of 12 wt.%, PMMA particle size of 150 μm, and PMMA particle content of 25 wt.%. Confirmation tests on the optimal parameters revealed improved results as well as some minor errors and deviations, indicating that the chosen parameters are critical for controlling the compressive properties of Al composites.

## 1. Introduction

Due to the unique properties of porous metals, their usage in the automobile industry, aerospace manufacturing, and other industries is progressively growing [1,2]. These properties include low relative density, high energy absorption capability, and adequate sound and heat insulation [3]. To improve the mechanical and physical properties, particularly higher strength of porous metals, the development of composite materials is highly required. Several strategies for improving the mechanical properties of porous Al by inclusion of reinforcement particles ex situ or in situ have been used. As a result, many researchers fabricate and studied porous composite with reinforcing particles such as SiC, Al_2_O_3_, B_4_C, and CNTs [4,5,6,7,8,9]. One such reinforcement, the diamond particles, has been used by several researchers in solid composites [10,11].

Diamond is an inert material with unique properties, such as the highest thermal conductivity, high hardness, and high strength. These properties are the most interesting for the objectives of this project. Diamond is an inert material with exceptional properties such as high strength, hardness, and thermal conductivity. At room temperature, diamond has the superior thermal conductivity of any existing material (k = [800–2000] W m1 K1), while exhibiting relatively low thermal expansion [12]. Considering the high strength of diamond and other properties, it can potentially improve the properties of porous composites. Further taking into account the applications in aerospace and automotives, it can be used as fillers in the crash box and other components to reduce the weight and prevent damage during accidents. As the evolution of safer materials may cost higher but their requirement is crucial. Keeping in view the safety of the passengers and expensive car components, such fillers can serve the purpose.

One of the biggest challenges associated with the use of diamond particles as reinforcement is their poor wettability resulting in weak interface between diamond and metal matrix. Several methods for improving diamond wettability have been investigated. The interfacial bonding of diamonds with metal matrix have been improved by surface treating or by applying coating or functionalized with high thermal conductivity metals or alloys such as Cu, Ag, and Al [13,14]. Another technique is to form a carbide layer between the diamond and metal interfaces to improve diamond wettability, for example, by using tungsten or titanium coating [13]. However, for their reinforcement in the porous Al composites, it is still in its infancy. 

One of the traditional methods employed for the development of advanced materials is powder metallurgy techniques. For the production of porous metal composites, the powder metallurgy technique using space-holder material as porous media is relatively simpler and controllable method [7,15,16,17]. The process steps generally are as follows: (1) mixing metal powder with reinforcing particles and space-holder material, (2) compression of prepared mixtures, (3) removal of space-holder material from the sample, and (4) sintering solid and liquid state [18,19]. The use of PMMA particles as space-holder material was considered to be the most beneficial [20,21]. The use of PMMA particles in porous Mg composite revealed the formation of spherical pores that replicate the shape and size of PMMA particles thus have better control on porosity [22]. Furthermore, their content and size also influence the properties of resultant material. Therefore, the present work is dedicated to studying the effect of different PMMA particle content and size and wt.% of Ti-coated diamond particles reinforcement on the microstructure and compressive properties of porous Al composites.

The study deals with development of porous Al composites with Ti-coated diamond reinforcing particles at different weight percentages using different content and size of PMMA space-holder particles. The powder metallurgy method technique in combination with Taguchi design of experiment was employed to fabricate the composites. According to Taguchi DOE, the porous composites for nine sets of parameters were evaluated. The pore morphology and elemental analysis of the porous Al composites was evaluated using SEM images and EDX. In addition, the mechanical properties of the porous composites were measured via compression testing and the plateau stress and energy absorption capability are determined. The effect of diamond content, PMMA content, and PMMA size on the compressive properties were then analyzed, experimentally and statistically.

## 2. Methodology

### 2.1. Experimental Procedure

#### 2.1.1. Fabrication of Porous Al Composites

Table 1 shows the composition of alloy matrix, reinforcement, and space holder content. The Al, Mg, tin, Cu, and boron powder (supplied by Sigma-Aldrich Sdn Bhd, Selongor, Malaysia) with the particle size of 45, 10, 45, 75, and 10 µm and purity of 99.9, 99.9, 99.5, 99.5, and 99.5% respectively were utilized as matrix materials. The diamond particles of particle size 45 and purity 99.5% were used as reinforcement and were received as Ti-coated by vacuum vapor deposition technique. While PMMA of purity 99.9% were added as space holders with varying particle content and particle size.

**Figure 1:**
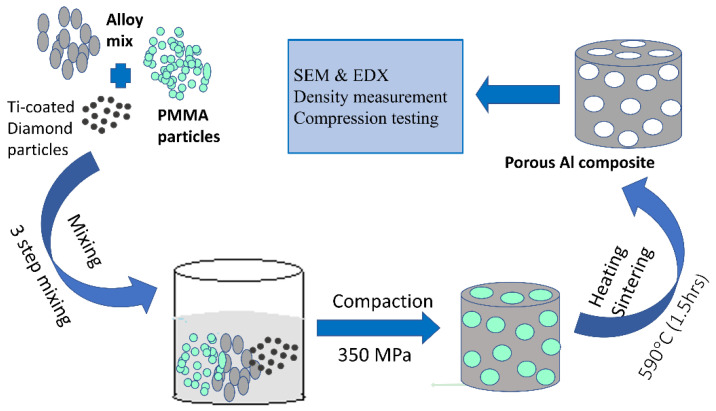
Fabrication process of porous Al composites.

The powder metallurgy technique was employed to fabricate the porous Al composites as shown in Figure 1. Initially, the blending process was carried out in three steps. In the first step, the alloy matrix powder containing AL, Mg, Sn, Cu, and B were mechanically blended at 300 rpm for 24 h in a ball mill with ball to powder ratio of 10:1, then the alloy matrix powder mix was added to Ti- coated diamond 800 rpm for 2 h particles using a shaker followed by mixing the resultant powder with PMMA particles at 800 rpm for 2 h at the heating rate of 2 °C/min. Prior mixing PMMA particles were mixed with CLE safe oil as a binder to bind them with metallic powder. The mixed powders were compacted uniaxially at the pressure of 350 MPa, followed by heat treatment according to the temperature required for the elimination of each space holder at 450 °C for 1 h, and then the samples were sintered at 590 °C for 1.5 h under an argon atmosphere using tubular furnace.

#### 2.1.2. Characterization

The total porosity (P) and relative density were determined from the density measurements by using the Archimedes method with the distilled water impregnation [23], using the following equation:

(1) $$R_{d}=\frac{\mathrm{Bulk}\;\mathrm{density}}{\mathrm{Material}\;\mathrm{density}}$$

where R_d_ represents the relative density, bulk density is the density calculated using Archimedes method, and material density is the density of Al matrix (2.7 g/m^3^).

Pore-morphology was characterized using scanning electron microscope (SEM) of JEOL JSM-6300F from Austin, TX, USA and the elemental analysis was carried out using electron dispersive X-ray (EDX) equipped with SEM. The compressive strength measurements were carried out according to the ASTM E9 standard [24] with a fixed crosshead speed of 0.5 mm/min at room temperature and load cell of 30 kN (Dartec model3500 universal testing machine (Selongor, Malaysia). The area under the stress–strain curves determined the energy absorption capacity (W) of the resulting porous Al composites using the following equation [25].

(2) $$W=\int_{0}^{\varepsilon }\sigma \;d\varepsilon$$

where σ and ε are the compression stress and strain, respectively.

### 2.2. Taguchi’s Design of Experiments (DOE)

Design of experiments deals with evaluation of factors that are held responsible for the control of a parameter or a group of parameters by conducting controlled tests and to estimate the best processing condition. DOE is helpful in investigating all possible combinations or a part of possible combinations. This is one of the valuable tools that helps in optimizing and better understanding the factors that aid in reducing material, energy, cost and time. Taguchi’s design of experiment includes orthogonal arrays and signal-to-noise ratios (S/N) concepts for analysis of data and prediction of optimum results. The S/N ratio, or signal-to-noise ratio, is the log function of the output of an analysis and is the prime function for optimization in a static problem. It is the product of experiments and is determined by a minimum number of experiments. Generally, in static problems the three S/N ratios for optimization are Smaller the better, Larger the better, and Nominal the best and based on the experiment it may be selected. In this study, the three input factors influencing the compressive properties such as diamond content, PMMA particle size, and PMMA particle content each with three levels were selected. The Taguchi model with three factors and three levels were chosen and thus orthogonal array L9 with nine rows and three columns were obtained. The three levels of the three parameters are shown in Table 2. The experiments were conducted based on the nine runs acquired from Taguchi model L9, and the objective of this model was to obtain higher compression properties (plateau stress and energy absorption capacity). The run order with the corresponding factors and levels are mentioned in Table 3. Upon the three S/N ratio characteristics larger the better was chosen for analysis. This S/N ratio was the measure of deviation of the quality characteristic from the value desired.

## 3. Results and Discussion

This experimental plan seeks to identify the critical factors and their interactions to attain the best compressive properties (plateau stress and energy absorption capacity). The porous Al composites were fabricated for nine sets of parametric combinations as per Taguchi orthogonal array and their compressive properties were recorded as shown in Table 3.

### 3.1. Morphology

**Figure 2:**
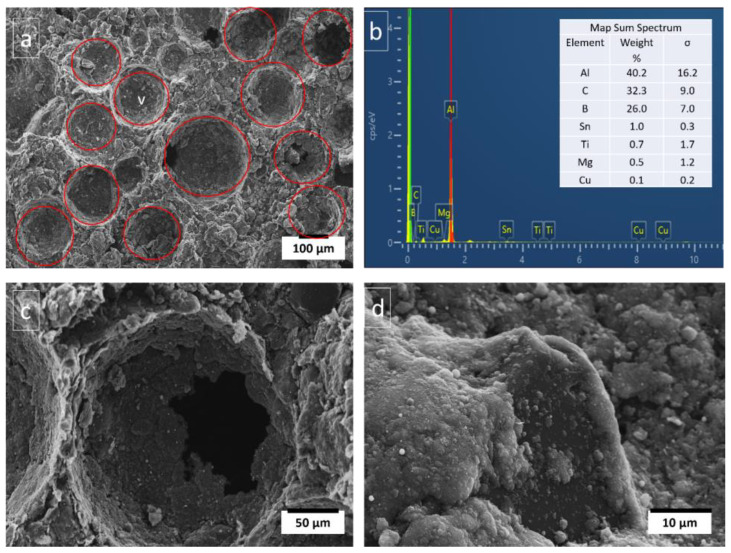
Microstructure of porous Al composite, (**a**) pore distribution and shape, (**b**) EDX of composite cross section, (**c**) pore cell and pore connectivity, and (**d**) well bonded diamond particle.

The cross-sectional view of porous Al composite as shown in Figure 2a exhibits two types of pore structures: macropores and micro-pores. Macro-pores are the desirable pores that were formed using PMMA particles as space holders, which decompose during sintering process leaving the pores behind. These are spherical shaped and have almost similar size as that of PMMA particles as shown in Figure 2a. This indicates that PMMA particles can tailor the pore size and shape, thus can control porosities of porous Al composites. Moreover, it has been observed that the presence of irregular pores causes difficulty in prediction of mechanical properties. The benefits of spherical shaped pores as shown in Figure 2a, include homogeneous pores, regular shape and size, and easier prediction of mechanical properties theoretically [26]. Jiang et al. investigated the mechanical properties of porous composites using spherical and angular shaped carbamide particles as space-holders. The porous composite with spherical carbamide has significantly better mechanical properties than porous composite with angular carbamide [15]. Moreover, the presence of rounded corners in spherical porosities contributes to reducing stress concentration. While micro-pores are the unwanted pores that were found in the macropore walls and struts. The macropores have pore connectivity as evident from Figure 2c. When the space holders slide with each other during compaction process, connected pores are formed after sintering. These connected pores aid in proper decomposition of the space holder during the sintering process [27].

Table 4 show the density and porosity of the porous Al with varying Ti-coated diamond (4,8 and 12 wt.%) and PMMA (20, 25 and 30 wt.%) and PMMA particle size (75, 125, and 150 µm). The density of porous Al composites initially increases insignificantly upto 25 wt.% followed by decrease with increase in PMMA particle size at constant diamond content of 4 wt.%. of, on the contrary the porosities increased. However, with increase in diamond content, the densities decreased, while porosities increased. This can be due to availability of insufficient alloy metal mix to fill the micropores formed as a result of higher Ti-coated diamond content [28]. Moreover, with increase in PMMA particle size the densities increased and on the contrary the porosities decreased. The presence of larger sized PMMA particles forms thicker cell walls and less cracks are formed as a result in comparison to small sized particles with weaker cell walls.

### 3.2. Effect of Diamond Particle Content

The potential role and proper distribution of reinforcing particles in the matrix allow composites to exhibit higher strength as compared to monolithic [29,30]. The uniform distribution of reinforcement particles in the matrix (cell wall) is critical for improving mechanical properties. Mostly, the reinforcing particles when added to the composites, get accumulated at the grain boundaries in powder metallurgy technique [31]. This leads to improvement in the properties such as yield strength, elastic modulus, and energy absorption capacity. In addition, more the uniformity of the cell wall, the greater is the strength of cell walls and the mechanical properties of the porous composites [32]. The intact and perfect cell wall with uniform dimensions influences the strength and physical properties of the porous materials [33]. 

**Figure 3:**
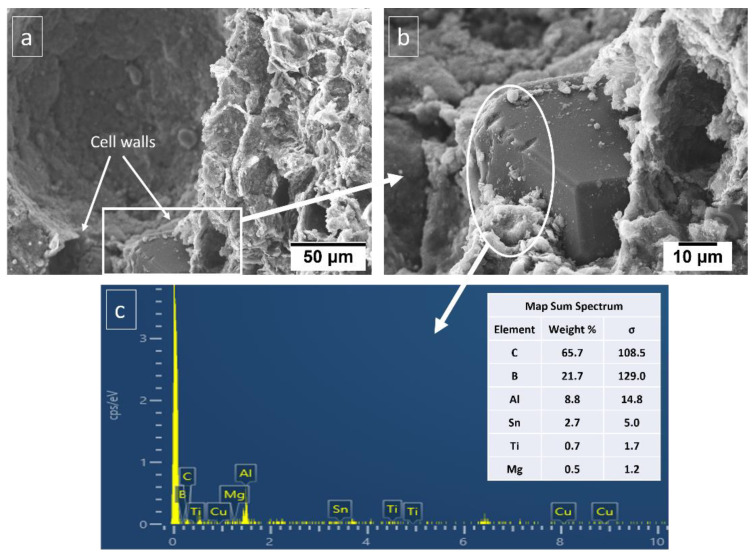
SEM images of (**a**) cell wall, (**b**) diamond particle in the cell wall and (**c**) EDS of material adhered to diamond particle in sintered porous Al composite.

It can be seen from Table 3 that the plateau stress of the porous Al composites increased with the increase in the wt.% of Ti-coated diamond particle. The energy absorption capacity of the composites also increased significantly with the increase in Ti-coated diamond particle content. In particular, the porous Al composites with porosity 20–30% with 4 wt.% of Ti-coated diamond exhibited plateau stress ranging from 24.8 to 26.37 MPa and for 8 wt.% of Ti-coated diamond content the plateau stress ranged from 29.86–36.68 MPa. However, the highest values were attained at 12 wt.% of Ti-coated diamond content in the range of 26.78–40.2 MPa. Similarly, the porous Al composites with porosity (20–30%) with 4 wt.% of Ti coated diamond showed energy absorption capacity of 4.88–6.78 Mj/m^3^ and the composites with 8 wt.% of Ti-coated diamond content displayed energy absorption capacity in the range of 7.93–10.75 Mj/m^3^. The best energy absorption capacity of the range 7.55–13.66 Mj/m^3^ was obtained for porous composites with 12 wt.% of Ti-coated diamond content. During the compression tests, Ti-coated diamond particles in the cell walls resist stress until their critical value is reached, when fracture occurs near or at the cell boundary. Moreover, the presence of Ti-coated diamond particles in the cell walls increases the cell wall thickness, as shown in Figure 3, the presence of diamond in the cell walls, and thus offers greater bending resistance. Moreover, Figure 3a,b shows that the matrix material held diamond particles so strongly that even when cross sectioned it stayed attached to the matrix and was not pulled out. This strong bonding is due to affinity of Ti coating with Al matrix and also due to the presence of sintering metal additives as evident from EDS in Figure 3c.

### 3.3. Effect of PMMA Particle Size

**Figure 4:**
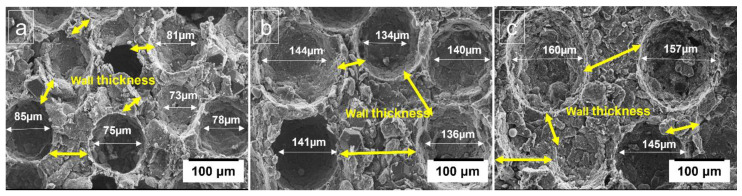
Pore size and wall thickness of pores in porous Al composites with varying PMMA particle size, (**a**) 75 µm, (**b**) 130 µm, and (**c**) 150 µm.

The porous composites replicate the shape and size of space holders, which is important in controlling porosity and pore size [22]. It allows the predictability and reproducibility of mechanical properties. The spherical structured cells (pores) and uniform distribution of the cell walls were obtained by adding the space holder particles of varying size, as evident from Figure 4. Due to uniform pore distribution in the porous structure, the strength of porous Al composite improved [34,35].

Table 3 shows that the composites with PMMA particle size of 150 µm exhibited higher plateau stress values than composites with smaller particle sizes (75 µm and 130 µm). The cell wall has a significant impact on overall load capacity. The smaller pores result in the formation of finer solid framework while the larger the pore size form the thicker cell walls and have high compressibility, which increases with pore size [36]. To maintain porosity, as pore size decreases, the number of pores increases, resulting in a smaller cell edge thickness. Furthermore, the cell edges may be too small to supply enough melt, resulting in impairing the mechanical properties significantly [37]. Therefore, the plateau strength decreases. Similarly, this effect was also seen in case of energy absorption capacities, where maximum values were acquired from the composites with 150 µm particle size of PMMA. 

### 3.4. Effect of PMMA Content

**Figure 5:**
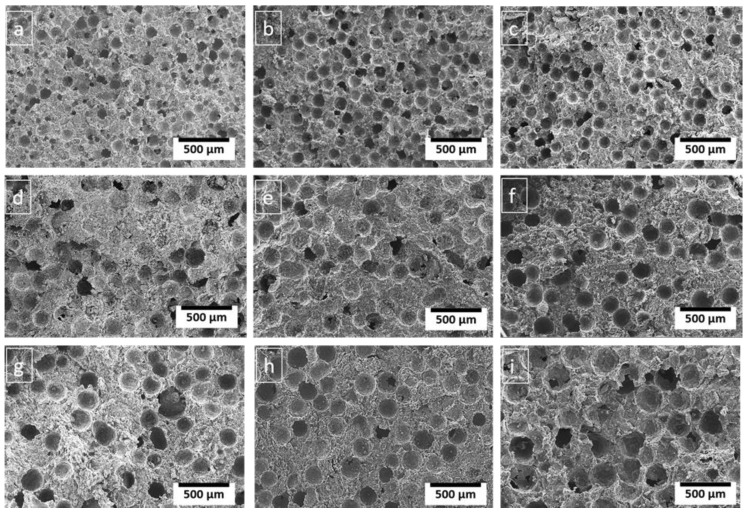
SEM micrography of porous Al composites at three particle size of PMMA viz 75 μm, 125 μm and 150 μm with varying PMMA particle content of (**a**,**d**,**g**) 20 wt.%, (**b**,**e**,**h**) 25 wt.%, and (**c**,**f**,**i**) 30 wt.% respectively.

Porosity influences the mechanical properties of porous composites [38] and attaining uniform dispersion could enhance mechanical response. The porosities can be easily controlled by proper selection of space holder content. In this study, the porosities of the porous composites were varied by using different contents of PMMA particles. The porosities in the porous Al composites are seen to depend on the PMMA content as evident from Table 3. The isolated pores were mostly found in porous Al composites containing 20% PMMA particles than in other wt.%. The interconnected pores were observed as the amount of PMMA particles increased, as shown in Figure 5b,c,e,f,h,j. Increasing the PMMA content from 20% to 30% increases the interconnected pore in porous Al composites. These interconnected pores aid in the better decomposition of the space holder during the sintering process [27]. As a result, the composites with 25 wt.% of PMMA content exhibited well-defined pore structure and least cracks as evident from Figure 5c,e,h and thus revealed greater compressive properties. The maximum values for plateau stress and energy absorption capacities ranged from 20.27–40.2 MPa and 5.12–13.66 respectively, at 25 wt.% of PMMA content.

### 3.5. Optimization Results

#### 3.5.1. Analysis of Means

**Figure 6:**
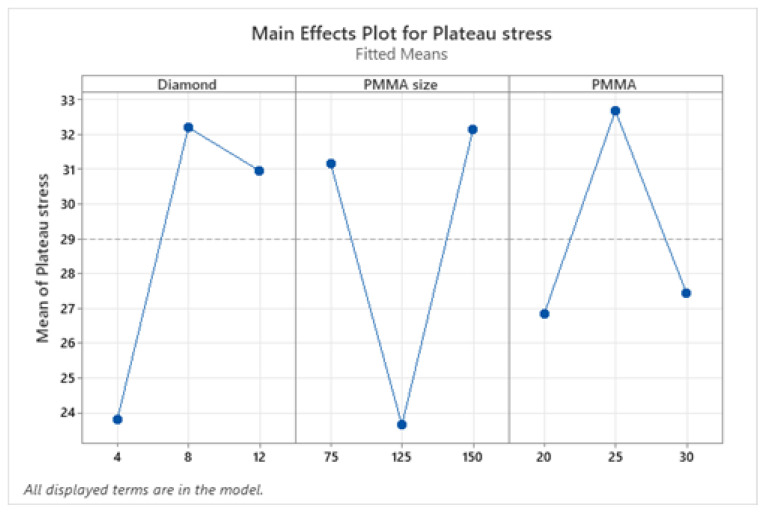
Mean response curve of plateau stress.

**Figure 7:**
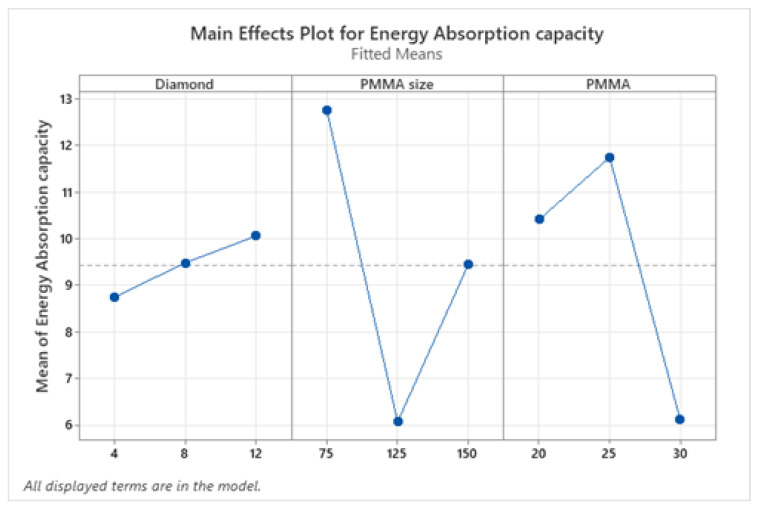
Mean response curve of energy absorption capacity.

The plateau stress and energy absorption capacity were regarded as response variables to improve the compressive properties. To increase their applicability, the values of plateau stress and energy absorption capacities should be maximized. As a result, the signal-to-noise (S/N) ratio for the response factors was determined by keeping the option “larger the better”. Figure 6 and Figure 7 show the main effect plot that explains the influence and impact of input factors on plateau stress and energy absorption capacity respectively. The results of compressive properties were analyzed using the MINITAB 18 software developed in 2017 in the University of Pennsylvania (Philadelphia, PA, USA) and the characteristic values were transformed to S/N ratio values. The mean of means and mean of S/N ratio graphs show that plateau stress increases with an increase in diamond weight percentage up to 8% and then reduces insignificantly. But for PMMA size, maximum plateau stress values are obtained at level 1 (75) and level 3 (150) and in case of PMMA content the plateau stress increases up to 25 wt.%, on further addition it decreases. Similarly, from means of means graph of energy absorption capacity, it increases with an increase in diamond content and maximum value was acquired at level 3(12 wt.%); for different sizes of PMMA particles it decreases then increases and maximum value was acquired at level 3(150); and for PMMA content it increased up to 25 wt.% beyond this it decreased, however the value at level 3 (30 wt.%) was found to be still higher than at lowest level 1 (20 wt.%) as shown in Figure 7.

#### 3.5.2. Analysis of Variance (ANOVA)

**Figure 8:**
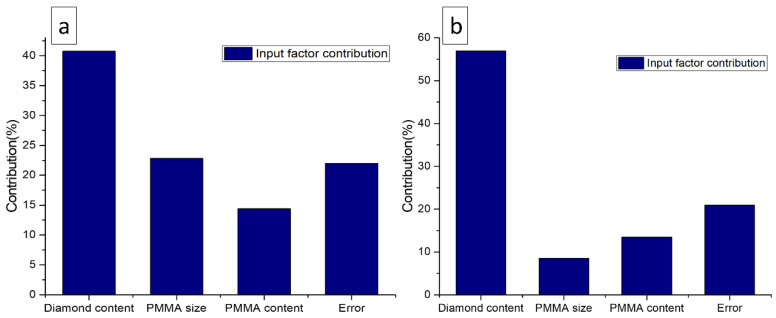
Percentage of the contribution of input factors on, (**a**) plateau stress, and (**b**) energy absorption capacity.

ANOVA serves to investigate the effect of parameters and their relationships by analyzing the mean square against response errors at predefined confidence levels. The ANOVA allows to find the effect of each factor on the overall variance of the results. The ANOVA results for the response factors; plateau stress and energy absorption capacity, are shown in Table 5 and Table 6 respectively. The analysis was conducted at a 10% significance level, which corresponds to a 90% level of confidence. Each ANOVA table contains a percentage contribution to total variation for each factor’s variable. The contribution of each factor that has a large impact on response factors (plateau stress and energy absorption capacity), as shown in Figure 8, is analyzed using the ANOVA table results. The diamond content shows the most significant (40.76%) effect on the plateau stress followed by PMMA size (22.85%) and PMMA content (14.41%), as evident from Table 5 and Figure 8a. Correspondingly, Table 6 and Figure 8b show that the diamond content highly affects the energy absorption capacity (56.96%) followed by a PMMA content (13.52%) and a PMMA size (8.54%). This could be a reason for the diamond content to influence the plateau stress and energy absorption capacity significantly, which is around 40.76% and 56.96% respectively.

As a result, the effect of diamond content in this study is maximum as compared to the PMMA size and PMMA content. The influence of the PMMA content is minimal (14.41%) in case of plateau stress, and PMMA size is minimal (8.54%) in case of energy absorption capacity. The addition of diamond particles increases the load bearing capacity of porous Al composites. As the modulus of the diamond particles is much greater than that of the matrix, the diamond particles bear the load directly by stress concentration. Moreover, during compression process, the additional stress concentration as a result of diamond particle jamming increases the particle’s load-carrying capacity, thereby improving compressive properties [39].

The linear polynomial model can be obtained from the analysis of variance, it defines the plateau stress and energy absorption capacity as a function of diamond content, PMMA size, and PMMA content. The greatest R2 predictions for plateau stress and energy absorption capacity were determined to be 94.49% and 99.18%, respectively, indicating that the model will predict new observations almost as well as it matches the sample data. Furthermore, the regression equation adequately explains this study.

### 3.6. Regression Analysis

The statistical model based on linear regression equations was acquired applying L9 orthogonal using MINITAB software. Based on each response factor, regression equations (or linear polynomial model) for plateau stress and energy absorption capacity were derived and presented as follows:

Plateau stress = 28.99 − 5.18 D_1_ + 3.21 D_2_+ 1.97 D_3_+ 2.18 P_s1_ − 5.33 P_s2_ + 3.15 P_s3_ − 2.14 P_c1_ + 3.69 P_c2_ − 1.55 P_c3_ (3)

Energy absorption capacity = 8.379 − 2.78 D_1_ + 1.10 D_2_ + 1.68 D_3_ − 0.04 P_s1_ − 1.03 P_s2_ + 1.08 P_s3_ − 1.12 P_c1_ + 1.47 P_c2_ − 0.35 P_c3_ (4)

where D is the diamond content, P_s_ is the PMMA particle size, and P_c_ is the PMMA content.

**Figure 9:**
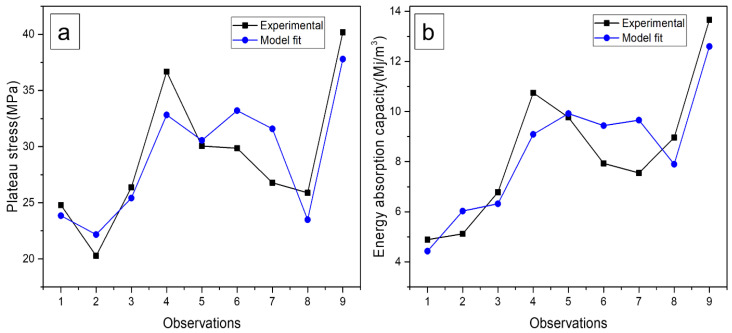
Evaluation of experimental and modal predicted (**a**) plateau stress and (**b**) energy absorption capacity.

The percentage deviations were obtained by calculating the response plateau stress and energy absorption values using linear regression Equations (3) and (4). The significant residual errors of each test, and the maximum errors of 1.12 and 1.39 (Table 7 and Table 8) were acquired for plateau stress and energy absorption capacity, respectively. To check the discrepancy in the experimental and model fit values, the data for each experiment run is illustrated in Figure 9. A rate of change of plateau stress and energy absorption capacity are produced by the device errors and processing errors. In addition, experimentation also relies on other factors including mixing parameters, and composition as the properties depend on these factors as well.

### 3.7. Response Optimization

**Figure 10:**
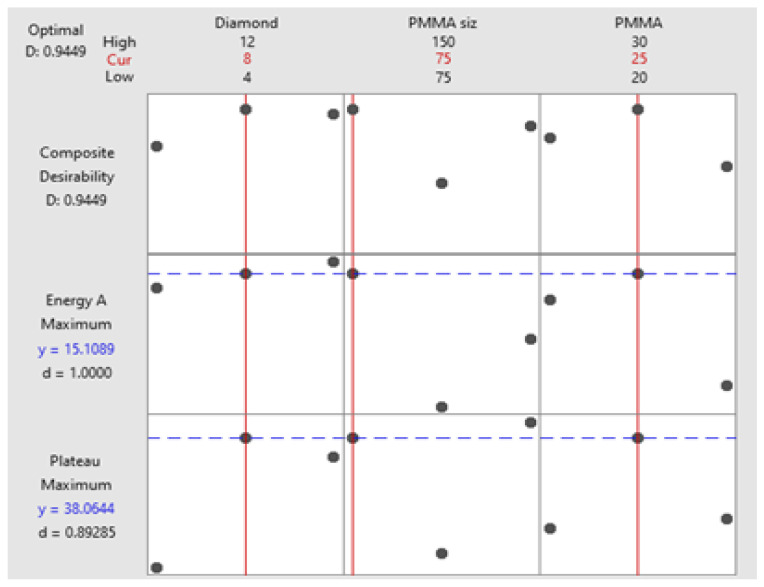
Optimization plot for plateau stress and energy absorption capacities.

The response optimization study was conducted to find the best results as shown in Table 9. The intention for conducting this study is to control the compressive properties of porous Al composites. In this study, the goal was to maximize the results in order to optimize plateau stress and energy absorption capacity. Table 9 shows the lower value of plateau stress, which can be considered the predicted value. The target and lower values have maximum and minimum variations, but this is impractical. Compression properties are also affected by alloy composition, crosshead speed during compression testing, and other factors. It may produce extremely high or low values. As a result, the fit values mentioned in Table 9 and shown in Figure 10 can be considered safe and reasonable values for plateau stress and energy absorption capacity, which can be attained with a combination of Ti-coated diamond content of 12 wt.%, PMMA particle size of 150 µm and PMMA particle content of 25 wt.%.

### 3.8. Interaction Plots

**Figure 11:**
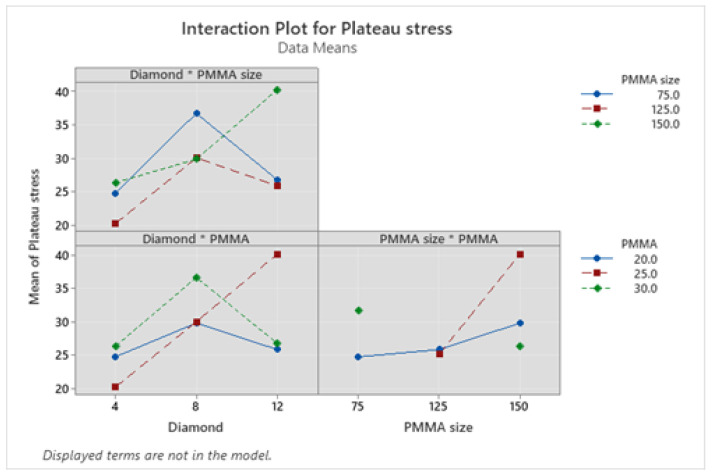
Interaction plots for plateau stress.

From interaction plots of plateau stress as shown in Figure 11, it can be observed that for diamond and PMMA size interaction, at PMMA size of 75 µm and 125 µm, the plateau stress increases up to 8 wt.% of diamond on further addition it decreases however for PMMA size of 150 µm, it increases with increase in diamond content from 4–12 wt.%. Similarly, for diamond and PMMA content interaction plot, the plateau stress values for PMMA content of 20 and 30 increases first from 4 to 8 wt.% of diamond content, on further addition it decreases. Even though for PMMA size of 25 wt.% it increases linearly on addition of diamond from 4 to 12 wt.%. Moreover, for PMMA size and PMMA content interaction plot, plateau stress for PMMA content of 20 and 25 increased on increasing the PMMA particle size, however for 30 wt.% it decreases.

**Figure 12:**
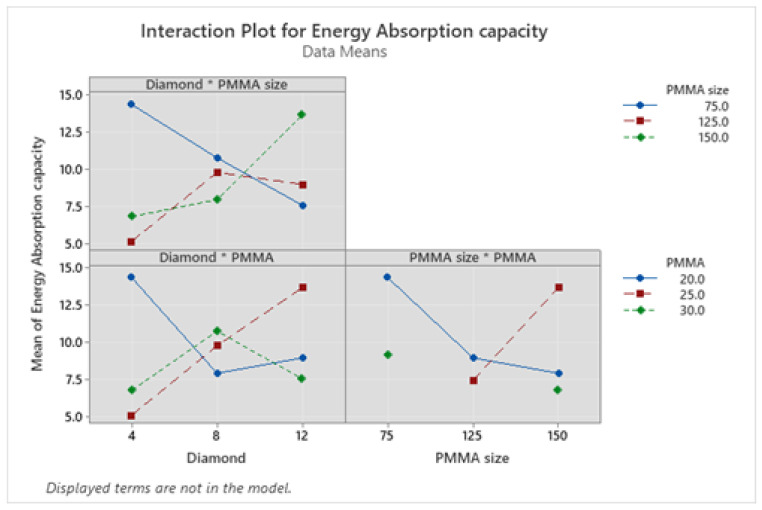
Interaction plots for energy absorption capacity.

Moreover, the interaction plots for energy absorption capacity in Figure 12 revealed that in the interaction plot of diamond and PMMA size, the energy absorption capacity increases first from 4 to 8 wt.% diamond content and on further addition it decreases in case of PMMA size of 75 µm and 125 µm, but for PMMA size of 150 µm, it increases with increase in diamond content from 4–12 wt.%. Moreover, for diamond and PMMA content interaction plot, the energy absorption capacity values for PMMA content of 30 wt.% increase first from 4 to 8 wt.% of diamond content, on further addition it decreases. Although for PMMA content of 20 and 25 wt.% it increases on addition of diamond from 4 to 12 wt.%, still the increase is more prominent in case of PMMA content of 25 wt.%. Moreover, for PMMA size and PMMA content interaction plot, energy absorption capacity for PMMA content of 25 wt.% increases on increasing the PMMA particle content but it decreases for 20 and 30 wt.% of PMMA content.

### 3.9. Contours Plot

**Figure 13:**
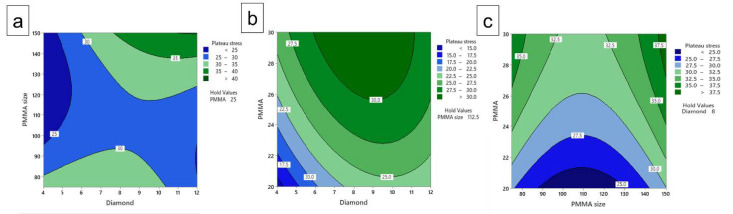
Contour plot of plateau stress for, (**a**) PMMA size vs. diamond content (**b**) PMMA vs. diamond content and (**c**) PMMA size vs. PMMA content.

**Figure 14:**
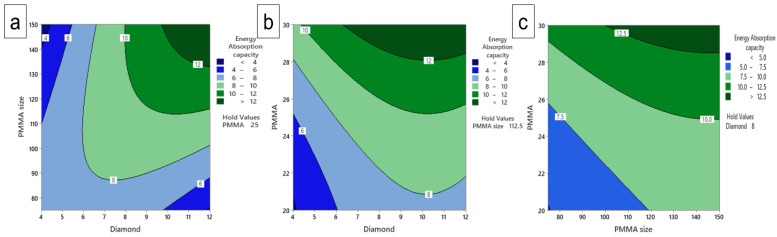
Contour plot of energy absorption capacity of, (**a**) PMMA size vs. diamond content (**b**) PMMA vs. diamond content and (**c**) PMMA size vs. PMMA content.

Comparison of the plateau stress and energy absorption capacity responses using contour plots in Figure 13 and Figure 14 shows three contour plots such as PMMA size versus diamond content, PMMA content contrasted with diamond content and PMMA content in competition with PMMA size based on the plateau stress and energy absorption capacity results. The contour plot variations were expressed as color variation, with different ranges for the individual colors based on the intensity of the experimental results. The contour plots show the influence in rise/decline of plateau stress and energy absorption capacity at withhold value of the PMMA content of 25 wt.% in Figure 13a and Figure 14a, PMMA size of 112.5 µm Figure 13b and Figure 14b, and diamond content of 8 wt.% in Figure 13c and Figure 14c, respectively. In combination with the parameters of Figure 12a, the plateau stress increases with an increase in diamond content and the influence of PMMA size showed increment followed by decrement of plateau stress. Figure 12b shows that the plateau stress increases with increase in PMMA content. However, for increased diamond content, it first increases then decreases. Moreover, Figure 12c shows the plateau stress increases while PMMA content increases, whereas for PMMA size, it decreases followed by increase with higher value for plateau stress. Moreover, it has been observed from Figure 12a that with an increase in PMMA size, the energy absorption decreases while as with increase in diamond content, it increases. Figure 13b reveals the energy absorption capacity increases with increase in PMMA size as well as diamond content. However, the diamond content has maximum influence thus providing higher value. Similar effect is seen in the contour plot of PMMA vs. PMMA size, also the value of energy absorption capacity is higher with the higher values of combined parameters (Figure 13c). The increase in compressive properties with increase in space holder content is attributed to the increase in the presence of well-defined pores that improve the compressive properties of the porous composites. Moreover, with the increase in space holder size, the cell size as well as cell wall thickness increases thereby increasing the compressive properties. Thus, with these contour results, it has been demonstrated that the selected inputs have an effect on changing the response of the present work.

### 3.10. Surface Plots

**Figure 15:**
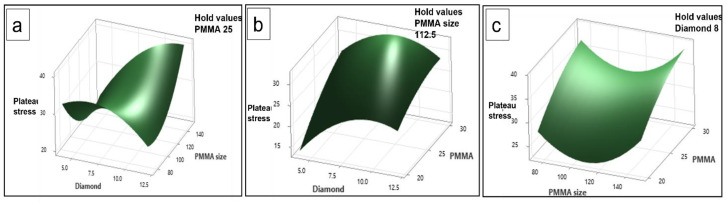
Surface plot of plateau stress vs. (**a**) PMMA size, diamond content, (**b**) PMMA and diamond content, and (**c**) PMMA size, PMMA content.

**Figure 16:**
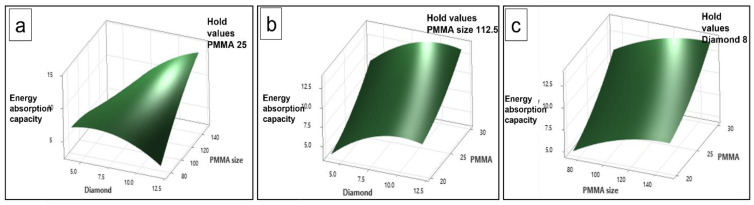
Surface plot of energy absorption capacity vs., (**a**) PMMA size, diamond content, (**b**) PMMA and diamond content, and (**c**) PMMA size, PMMA content.

Similarly, Figure 15 and Figure 16 show the surface plots comparison for plateau stress and energy absorption capacity as the three-in-one diagram. The results of plateau stress and energy absorption capacity are compared with two factors in each graph, including diamond content compared with PMMA size, PMMA content contrasted with diamond content, and PMMA size as opposed to PMMA content, respectively. Furthermore, results were extracted from some of the chosen input combinations for the contours and the maximum values were found with these data. These surface plots are the three-dimensional view of the contour plots, and they can observe the significant changes in a surface with numerical values of each combined parameter with one hold value. Figure 15 shows the plateau stress range that can be understood by the contours’ color, ranging from light to dark. Based on Figure 15 with the response of plateau stress, each values surface plot has hold values such as PMMA 25 in Figure 15a, which means that the obtained results of response will be varying by size and diamond and constant values are the hold values with these combination response showing the optimum results for the output value. Moreover, Figure 15b,c show the results considering one hold value and other parameters varying. Generally, the response value depends on the input parameters and their levels. With these surface plots, the phenomena of input parameters combination and three-dimensional variation for a suitable output can be realized. 

Similarly, the surface plots for energy absorption capacity have been illustrated in Figure 16. In these plots, the hold values are the same as in Figure 14 and the parameters combination after all the phenomenon of three-dimensional variation is completely changed. This is because the combination of input parameters behaves differently, therefore the surface is varied in a different way. However, with these results, it can be realized how significant is the impact of the input parameters and their level on the response values. Considering these kinds of studies will result in choosing the best possible combination of parameters to fit the values of the optimum response. 

### 3.11. Confirmation Test

**Figure 17:**
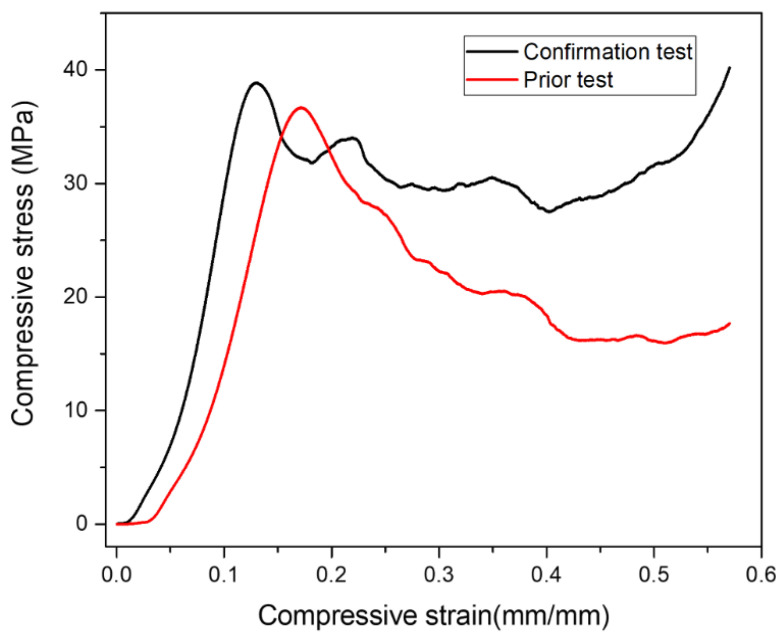
Comparison of confirmation test result at optimal input factors and maximum value obtained from Taguchi L9 runs.

The final step in the DOE approach is experiment confirmation. Following the investigation of the optimal test conditions, the confirmation was carried out with the optimal level of factors, diamond content of 12 wt.%, PMMA particle size of 150 m, and PMMA particle content. Finally, the obtained results were compared to the predicted results. Figure 17 depicts the stress–strain diagram showing the comparison of confirmation test result with the highest value obtained from Taguchi L9 runs demonstrated in Table 3. From Figure 17, it is clear that the confirmation test results exhibited the highest plateau stress, that was more stable as compared to the result obtained in the prior test.

Table 10 also shows the comparative results obtained with the best parameters. It was discovered that the experimental and predicted results were in close agreement. However, an error of 6.4% and 11.1% was observed in plateau stress and energy absorption capacity, respectively.

## 4. Conclusions

1. In this study, the effect of reinforcement content, space holder size, and space holder particle content on the compressive properties of porous Al composites using experimental, numerical, and optimization methods was investigated.
2. The developed composites exhibited a spherical porous structure with Ti-coated diamond particles distributed uniformly within the Al matrix alloy.
3. The densities and porosities also improved due to the presence of Ti-coated diamond particles that were well bonded with the Al matrix alloy, revealing improved wettability and also by the inclusion of additives like Mg, Sn, Cu, and B, which aided in liquid sintering.
4. The higher values of plateau stress and energy absorption were obtained at the diamond content of 12 wt.%, PMMA particle size of 150 μm, and PMMA particle content of 25 wt.%.
5. The effect of input factors on compressive properties was investigated by applying statistical and regression analyses, model prediction, contour, and surface plots. The linear regression equation values were compared to the results of the experimental tests. The response optimized results included Ti-coated diamond content of 12 wt.%, a PMMA particle size of 150 m, and a PMMA particle content of 25 wt.%.
6. Finally, the findings were validated by running the confirmation test under optimal conditions. With the lowest percentage deviation in plateau stress and energy absorption capacity, the model was found to be reliable and significant.
7. The findings of the present study agree well with the −6.4% and 11.1% marginal discrepancy in plateau stress and energy absorption capacity values, respectively. This difference can be attributed to the other factors, such as processing parameters and varying compositions, that also affect the compressive properties.

## Figures and Tables

**Table 1:** Composition of Ti-coated diamond reinforced porous Al composites.

**Alloy Matrix**	**Al**	**Mg**	**Sn**	**Cu**	**B**	**Reinforcement**	**Ti-Diamond**	**Space Holder**	**PMMA**
**Wt.%**	94	1	2	2	1		4, 8, 12		20, 25, 30

**Table 2:** Control factors (process parameters) and their levels.

Factors	Symbol	Unit	Level 1	Level 2	Level 3
Diamond content	A	wt.%	4	8	12
PMMA size	B	µm	75	125	150
PMMA content	C	wt.%	20	25	30

**Table 3:** L9 Orthogonal array layout with design factors.

S.no	Diamond (wt.%)	PMMA Size (µm)	PMMA (wt%)	Plateau Stress (MPa)	Energy Absorption Capacity (Mj/m^3^)
1	4	75	20	24.8	4.89
2	4	125	25	20.27	5.12
3	4	150	30	26.37	6.78
4	8	75	30	36.68	10.75
5	8	125	25	30.06	9.77
6	8	150	20	29.86	7.93
7	12	75	30	26.79	7.55
8	12	125	20	25.89	8.96
9	12	150	25	40.2	13.66

**Table 4:** Densities and porosities of porous Al composites for nine sets of parameters.

S.no	1	2	3	4	5	6	7	8	9
**Porosity (%)**	21	24	30	29	26	18	31	20	25
**Bulk density**	1.94	2.06	1.76	2.08	2	1.92	2	1.867	1.774
**Relative density**	0.72	0.76	0.65	0.77	0.74	0.71	0.74	0.69	0.66

**Table 5:** ANOVA variance table for plateau stress.

Source	DF	Seq SS	Contribution	Adj SS	Adj MS	F-Value	*p*-Value
Diamond	2	122.95	40.76%	122.95	61.47	1.85	0.350
PMMA size	2	68.91	22.85%	91.66	45.83	1.38	0.420
PMMA	2	43.46	14.41%	43.46	21.73	0.66	0.604
Error	2	66.29	21.98%	66.29	33.14		
Total	8	301.61	100.00%				

**Table 6:** ANOVA variance table for energy absorption capacity.

Source	DF	Seq SS	Contribution	Adj SS	Adj MS	F-Value	*p*-Value
Diamond	2	35.326	56.96%	35.326	17.663	2.72	0.269
PMMA size	2	5.300	8.54%	6.047	3.024	0.46	0.683
PMMA	2	8.388	13.52%	8.388	4.194	0.64	0.608
Error	2	13.009	20.97%	13.009	6.504		
Total	8	62.023	100.00%				

**Table 7:** Fits and diagnostics for all observations for plateau stress.

Obs	Plateau Stress	Fit	SE Fit	Resid	Std Resid	Del Resid
1	24.80	23.85	5.50	0.95	0.55	0.43
2	20.27	22.17	4.62	−1.90	−0.55	−0.43
3	26.37	25.42	5.50	0.95	0.55	0.43
4	36.68	32.83	4.62	3.85	1.12	1.30
5	30.06	30.56	4.62	−0.50	−0.15	−0.10
6	29.86	33.21	5.08	−3.35	−1.23	−1.79
7	26.79	31.59	4.62	−4.80	−1.40	−6.62
8	25.89	23.49	5.50	2.40	1.40	6.62

**Table 8:** Fits and diagnostics for all observations for energy absorption capacity.

Obs	Energy Absorption Capacity	Fit	SE Fit	Resid	Std Resid	DFITS
1	4.89	4.43	2.43	0.46	0.60	1.4955
2	5.12	6.03	2.05	−0.91	−0.60	−0.6289
3	6.78	6.32	2.43	0.46	0.60	1.4955
4	10.75	9.09	2.05	1.66	1.09	1.6322
5	9.77	9.92	2.05	−0.15	−0.10	−0.0922
6	7.93	9.44	2.25	−1.51	−1.26	−3.6402
7	7.55	9.66	2.05	−2.11	−1.39	−7.2209
8	8.96	7.90	2.43	1.06	1.39	17.1717
9	13.66	12.60	2.43	1.06	1.39	17.1717

**Table 9:** Response optimization: composition and characteristics.

Response			Goal	Lower	Target	Upper	Weight	Importance
Energy Absorption capacity			Maximum	4.89	13.66		1	1
Plateau stress			Maximum	20.27	40.20		1	1
**Solution**								
**Solution**	**Diamond**	**PMMA size**	**PMMA**	**Energy Absorption capacity Fit**		**Plateau** **Stress** **Fit**		**Composite** **Desirability**
1	12	150	25	12.6029		37.7998		0.879515
**Variable Settings**								
Diamond				12				
PMMA size				150				
PMMA				25				
**Response**		**Fit**	**SE Fit**	**95% CI**		**95% PI**		**Fit**
Energy Absorption capacity		12.60	2.43	(2.13, 23.08)		(−2.57, 27.77)		
Plateau stress		37.80	5.50	(14.16, 61.44)		(3.56, 72.04)		

**Table 10:** Comparison of confirmation test with predicted values.

Responses	Prediction	Experimentation	Error (%)
Plateau stress (MPa)	37.79	40.21	−6.4
Energy absorption capacity (Mj/m^3^)	12.60	11.20	11.1

## Data Availability

Not applicable.
